# Narrative Preparedness; A Response to Recent Commentary

**DOI:** 10.34172/ijhpm.9001

**Published:** 2025-02-19

**Authors:** Eivind Engebretsen, Mona Baker

**Affiliations:** Sustainable Health Unit, University of Oslo, Oslo, Norway.

 If the recent pandemic has taught us anything, it is that culture and the narratives people subscribe to play a crucial role in determining the extent to which health evidence is understood, accepted, and acted upon. Despite this, we remain slow to integrate these insights into health preparedness efforts.

 Catherine Grant^1^ provides important insights in her comment on our paper on narrative preparedness.^2^ She argues that a holistic, context-driven strategy that prioritises building trust with communities in tackling the new challenges arising from contemporary pandemics and epidemics is crucial for enhancing future preparedness efforts, and supports her argument with an illuminating example from the West Africa Ebola outbreak in 2014-2016. One of the key transmission pathways of Ebola was unsafe burials. Burials subsequently became medicalised and regulated, when previously they were organized by communities and involved performing important rituals believed to enable the dead person to accede to the “village of the ancestors” where they would reunite with the dead. This important cultural narrative meant that people resisted the response teams and attempted to adhere to traditional funerary practices, which exacerbated the epidemic and led to some internments becoming “super-spreading” events. Alternative burial practices then had to be organized in consultation with local communities, in an effort to understand and accommodate cultural beliefs and needs surrounding burials and ultimately to ensure that these practices are adhered to by local populations. As Richards points out, “epidemiologically safe burial is unsafe from a social and spiritual perspective” (p. 52).^3^ This marked a pivotal moment as epidemic response agencies started to recognise that engaging with local social dynamics and contexts can mitigate the extra costs and harm associated with “context-blind” interventions.

 Grant suggests supplementing Fisher’s narrative paradigm with a specific model developed by Leach to address vaccine hesitancy in Africa. This model, she argues, expands upon Fisher’s narrative paradigm by emphasising socio-political and historical contexts, particularly in African settings. It considers how colonial legacies, political trust, and local experiences shape vaccine perceptions, providing a more nuanced understanding of the factors influencing public responses. By shifting the focus from narratives to anxieties, Leech’s model is more able to capture and account for emotional and cognitive responses to vaccines.

 However, Grant’s most important contribution, in our view, is that she pinpoints the challenge of combining narrative preparedness with traditional health preparedness. She stresses that this challenge calls for transdisciplinary approaches and collaboration between public health experts and social scientists. This, moreover, “requires a cultural change within health institutions that have traditionally prioritised quantitative data over qualitative insights.” Health professionals, academics and policy-makers have learned that trust and engagement with local communities, their culture and beliefs are crucial in addressing the challenge of a new pandemic. However, they fail to understand that this requires a cultural shift within the health academic communities themselves.

 Measuring a country’s ability to respond rapidly to emerging infectious disease threats has traditionally been a primarily quantitative endeavor. As Tan and colleagues observe,^4^ existing monitoring and evaluation systems—such as the Global Health Security Index and the Epidemic Preparedness Index—focus on structural aspects of health systems that can be readily counted or measured, including laboratory infrastructure, financing, surveillance, and emergency response operations. Although these dimensions are undeniably important, they fail to capture equally critical factors—governance, cooperation, and collaboration—that are not as easily quantified.

 At the same time, unless medical science acknowledges that it, too, constitutes a cultural framework, it is unlikely to recognize other ways of thinking as legitimate. In our article, we argue that equating rationality solely with scientific rationality—and labeling everything else as irrational—stifles communication with alternative narrative rationalities. This is precisely why a narrative preparedness approach is so vital. However, scientific rationality can also impede wider acceptance of narrative preparedness by dismissing it as a “soft” or less important dimension, overshadowed by what are deemed the more “hard” or technical elements of preparedness work.

 In an age of widespread misinformation, it is important to clarify that acknowledging science’s cultural and historical dimensions is not equivalent to denying its value or embracing relativism. On the contrary, we believe that science and science communication are strengthened by recognizing that science is part of a broader narrative—one that shapes both the strong reliance some people place on it and the skepticism of others towards it. Far from being a threat, this narrative awareness is integral to the scientific endeavor.

## Ethical issues

 Not applicable.

## Conflicts of interest

 Authors declare that they have no conflicts of interest.
